# Socioeconomic, environmental and lifestyle factors associated with gestational diabetes mellitus: A matched case-control study in Beijing, China

**DOI:** 10.1038/s41598-018-26412-6

**Published:** 2018-05-25

**Authors:** Xianming Carroll, Xianhong Liang, Wenyan Zhang, Wenjing Zhang, Gaifen Liu, Nannette Turner, Sandra Leeper-Woodford

**Affiliations:** 1 Department of Community Medicine, Mercer University School of Medicine, Macon, USA; 2 Department of Neurology, Beijing Tiantan Hospital, Capital Medical University, Beijing, China; 3 China National Clinical Research Center for Neurological Diseases, Beijing, China; 4 Department of Obstetrics and Gynecology, Beijing Chaoyang District Hospital of Maternal and Child Health, Beijing, China; 5 Department of Obstetrics, Beijing Chuiyangliu Hospital, Beijing, China; 6 Department of Public Health, Mercer University College of Health Professions, Atlanta, USA; 7 Department of Biomedical Sciences, Mercer University School of Medicine, Macon, USA

## Abstract

Gestational diabetes mellitus (GDM) is a common health problem during pregnancy and its prevalence is increasing globally, especially in China. The aim of this study was to investigate socioeconomic, environmental and lifestyle factors associated with GDM in Chinese women. A matched pair case-control study was conducted with 276 GDM women and 276 non-GDM women in two hospitals in Beijing, China. Matched factors include age and pre-pregnancy body mass index (BMI). GDM subjects were defined based on the International Association of Diabetes Study Group criteria for GDM. A conditional logistic regression model with backward stepwise selection was performed to predict the odds ratio (OR) for associated factors of GDM. The analyses of data show that passive smoking at home (OR = 1.52, p = 0.027), passive smoking in the workplace (OR = 1.71, p = 0.01), and family history of diabetes in first degree relatives (OR = 3.07, p = 0.004), were significant factors associated with GDM in Chinese women. These findings may be utilized as suggestions to decrease the incidence of GDM in Chinese women by improving the national tobacco control policy and introducing public health interventions to focus on the social environment of pregnant women in China.

## Introduction

Gestational diabetes mellitus (GDM) is glucose intolerance that first appears during pregnancy, and is usually associated with short- and long-term health problems such as prenatal morbidity^1–3^ and development of type 2 diabetes in the years after pregnancy^4,5^. Children born to women with GDM have an increased risk of obesity and diabetes in childhood and early adulthood^6–8^. Some of the reported risk factors for GDM include advanced maternal age, pre-pregnancy obesity, previous delivery of a newborn with congenital malformations such as macrosomia, previous history of GDM, cesarean section, and a family history of diabetes in first degree relatives^9–12^. Other possible risk factors have been proposed, including gestational weight gain, multi-parity, exposure to tobacco smoke, alcohol consumption, physical inactivity, consumption of sugar-sweetened beverages, and socioeconomic factors such as education, occupation, and household income^10,11,13^.

In the past two decades, China has witnessed rapid lifestyle and socioeconomic changes with increasing westernization, characterized by changes in behaviour including exposure to tobacco smoke, increased alcohol consumption, changes in dietary choices, and physical inactivity^14^. These factors have been implicated in the rapidly increasing prevalence of GDM, which ranges between 6.8% and 10.4% in pregnant women in China^15,16^, and even rose to higher levels (19.7%) in Beijing^17^. Whether these changes in lifestyle patterns and other associated risk factors play roles in the increasing prevalence of GDM in China has not been investigated.

The aim of this study was to investigate the association between socioeconomic, environmental and lifestyle factors with GDM among pregnant Chinese women in Beijing. The study hypotheses are: (1) when compared to pregnant women without GDM, pregnant women who develop gestational diabetes are more likely to be in the lower socioeconomic status (SES) as measured by education, occupation and household income; (2) when compared to pregnant women without GDM, pregnant women who develop GDM are more likely exposed to environmental tobacco smoke (ETS), or choose unhealthy lifestyles as measured by tobacco smoking, alcohol consumption, physical activity and diet.

## Results

### Distribution of matched factors and socioeconomic factors in GDM cases and controls

The analysis included 276 women in the GDM case group and 276 women in the non-GDM control group matched by age and pre-pregnancy body mass index (BMI). In Table 1, there was no statistical difference either on age or pre-pregnancy BMI between the two groups: mean age was 29.31 ± 4.30 years in the case group and 29.32 ± 4.30 years in the control group; mean pre-pregnancy BMI was 23.90 ± 3.37 kg/m^2^ in the case group and 23.92 ± 3.32 kg/m^2^ in the control group.

**Table 1:** Distribution of matched factors and socioeconomic factors in GDM: comparison of GDM cases and controls.

Characteristics	GDM cases n = 276 (%)	Controls n = 276 (%)	*p-value	OR (95%CI)	**p-value
**Matched factors**					
Age (years)	29.31 ± 4.30	29.32 ± 4.30	0.972	—	—
Pre-pregnancy BMI (kg/m^2^)	23.90 ± 3.37	23.92 ± 3.32	0.935	—	—
**Socioeconomic factors**					
Ethic group			0.554		
Han nationality	261 (94.6)	264 (95.7)		1 (Reference)	
Other ethics	15 (5.4)	12 (4.3)		1.27 (0.58–2.80)	0.550
Educational level			0.002		
Low (≤9 years)	54 (19.6)	81 (29.3)		1 (Reference)	
Middle (9–12 years)	95 (34.5)	106 (38.4)		1.35 (0.86–2.12)	0.194
High (>12 years)	126 (45.8)	89 (32.2)		2.13 (1.36–3.34)	0.001
Occupation			0.198		
Housewife	124 (44.9)	143 (51.8)		1 (Reference)	
Manual labor	53 (19.2)	53 (19.2)		1.18 (0.74–1.88)	0.491
Office worker	41 (14.9)	40 (14.5)		1.20 (0.73–1.96)	0.481
Other type	58 (21.0)	40 (14.5)		1.69 (1.05–2.72)	0.032
Household income			0.013		
<3000 Yuan/month	60 (21.7)	79 (28.6)		1 (Reference)	
3000–5999 Yuan/month	108 (39.1)	118 (42.8)		1.17 (0.75–1.81)	0.489
6000–8999 Yuan/month	61 (22.1)	55 (19.9)		1.50 (0.90–2.53)	0.123
≥9000 Yuan/month	47 (17.0)	24 (8.7)		2.64 (1.43–4.86)	0.002
Marital status			0.055		
Married	263 (95.3)	271 (98.2)		1 (Reference)	
Single/Divorced/Widowed	13 (4.7)	5 (1.8)		2.60 (0.93–7.20)	0.069
Residency in Beijing			0.023		
≤5 years	92 (33.3)	118 (42.8)		1 (Reference)	
>5 years	184 (66.7)	158 (57.2)		1.57 (1.08–2.27)	0.018
Living condition			0.069		
House owner	63 (22.8)	46 (16.7)		1 (Reference)	
Rental/Living with parents/Other	213 (77.2)	230 (83.3)		0.67 (0.43–1.03)	0.067

*P-value was obtained from student t-test for continuous variables and chi-square test for categorical variables. **P-value was obtained from the multiple logistic regression model that simultaneously included socioeconomic factors.

In the descriptive analysis (Table 1), GDM was more likely to be found in the comparisons of educational level (p = 0.002), household income (p = 0.013), and length of residency in Beijing (p = 0.023). In Table 1, Odds ratio (OR) and 95% confidence interval (95% CI) of socioeconomic factors were presented for GDM cases and controls by multiple logistic regression analysis. The number of women who developed GDM was significantly higher in those who received more than 12 years of education when compared to those with less than 9 years of education (OR = 2.13, p = 0.001). We found a significant difference in number of women with GDM when comparing women who worked in other types of jobs with housewives (OR = 1.69, p = 0.032). We also found a significant difference in GDM when comparing women with household income ≥9000 Yuan/month and those with a household income <3000 Yuan/month (OR = 2.64, p = 0.002). Comparing the women who resided in Beijing for less than 5 years, those with residency longer than 5 years showed a significant difference associated with GDM (OR = 1.57, p = 0.018).

### Distribution of environmental and lifestyle factors in GDM cases and controls

In Table 2, the descriptive analyses showed that GDM subjects were more likely to be exposed to passive smoking at home from their husbands (p = 0.009) and passive smoking in their workplaces (p = 0.013). GDM subjects were also more likely to consume alcohol before pregnancy (p = 0.008) and consume alcohol during pregnancy (p = 0.036). The multiple logistic regression analyses also showed a significant association between passive smoking at home from the husband and GDM (OR = 1.58, p = 0.011), as well as passive smoking in the workplace and GDM (OR = 1.68, p = 0.009). Associations in the regression analyses were also found between alcohol consumption before pregnancy and GDM (OR = 1.77, p = 0.013), between alcohol consumption during pregnancy and GDM (OR = 1.76, p = 0.038) in Table 2.

**Table 2:** Distribution of environmental and lifestyle factors in GDM: comparison of GDM cases and controls.

Characteristics	GDM cases n = 276 (%)	Controls n = 276 (%)	*p-value	OR (95%CI)	**p-value
**Environmental tobacco smoke (ETS)**					
Passive smoking at home from husband			0.009		
Yes	105 (38.0)	76 (27.5)		1.58 (1.11–2.25)	0.011
No	171 (62.0)	200 (72.5)		1 (Reference)	
Passive smoking from other family member			0.129		
Yes	50 (18.1)	37 (13.4)		1.50 (0.91–2.46)	0.109
No	226 (81.9)	239 (86.6)		1 (Reference)	
Passive smoking in the workplace			0.013		
Yes	86 (31.3)	60 (21.9)		1.68 (1.13–2.49)	0.009
No	189 (68.7)	214 (78.1)		1 (Reference)	
**Lifestyle factors**					
Tobacco smoking			0.699		
Tobacco smoking before pregnancy	21 (7.6)	17 (6.2)		1.24 (0.66–2.36)	0.505
Tobacco smoking during pregnancy	10 (3.6)	8 (2.9)		1.27 (0.50–3.21)	0.620
Never	245 (88.8)	251 (90.9)		1 (Reference)	
Alcohol consumption before pregnancy			0.008		
Yes	66 (23.9)	41 (15.0)		1.77 (1.13–2.77)	0.013
No	210 (76.1)	233 (85.0)		1 (Reference)	
Alcohol consumption during pregnancy			0.036		
Yes	41 (14.9)	25 (9.1)		1.76 (1.03–3.01)	0.038
No	235 (85.1)	251 (90.9)		1 (Reference)	
Physical activity 60 minutes			0.173		
<3 days/week	144 (52.2)	128 (46.4)		1.28 (0.91–1.81)	0.162
≥3 days/week	132 (47.8)	148 (53.6)		1 (Reference)	
Sports (swim/dance/playing ball)			0.730		
Yes	17 (6.2)	19 (6.9)		0.89 (0.45–1.74)	0.732
No	259 (93.8	257 (93.1)		1 (Reference)	
TV viewing			0.807		
≥3 hours	38 (13.8)	40 (14.5)		0.94 (0.58–1.53)	0.803
<3 hours	238 (86.2)	236 (85.5)		1 (Reference)	
Sleeping hours			0.195		
<8	39 (14.1)	29 (10.5)		1.42 (0.84–2.39)	0.191
≥8	237 (85.9	247 (89.5)		1 (Reference)	
Fruit intake ≥3 times/day			0.349		
Yes	28 (10.1)	35 (12.7)		0.77 (0.45–1.32)	0.347
No	248 (89.9	241 (87.3)		1 (Reference)	
Sugar sweetened soft drink ≥3 times/day			1.000		
Yes	4 (1.4)	4 (1.4)		1.00 (0.25–4.00)	1.000
No	272 (98.6)	272 (98.6)		1 (Reference)	

*P-value was obtained from student t-test for continuous variables and chi-square test for categorical variables. **P-value was obtained from the multiple logistic regression model that simultaneously included environmental and lifestyle factors.

### Distribution of biological factors in GDM cases and controls

In Table 3, the data show that GDM subjects were more likely to have a family history of diabetes in first degree relatives (p < 0.001). The multiple logistic regression analysis showed that a family history of diabetes in first degree relatives was associated with GDM (OR = 3.22, p = 0.002) in Table 3.

**Table 3:** Distribution of biological factors in GDM: comparison of GDM cases and controls.

Characteristics	GDM cases n = 276 (%)	Controls n = 276 (%)	*p-value	OR (95%CI)	**p-value
**Biological factors**					
Menarche age			0.815		
<12 years old	9 (3.3)	10 (3.6)		0.88 (0.33–2.41)	0.796
≥12 years old	267 (96.7)	266 (96.4)		1 (Reference)	
Menstrual cycle (28 days)			0.857		
Abnormal	95 (34.4)	93 (33.7)		1.03 (0.73–1.47)	0.858
Normal	181 (65.6)	183 (66.3)		1 (Reference)	
Number of pregnancies			0.132		
≥2	168 (60.9)	185 (67.0)		0.77 (0.55–1.09)	0.139
0–1	108 (39.1)	91 (33.0)		1 (Reference)	
Number of live births			0.797		
≥1	155 (56.2)	158 (57.2)		0.95 (0.67–1.35)	0.789
0	121 (43.8)	118 (42.8)		1 (Reference)	
Number of miscarriages/abortions			0.496		
≥1	135 (48.9)	143 (51.8)		0.90 (0.65–1.24)	0.510
0	141 (51.1)	133 (48.2)		1 (Reference)	
Oral contraceptive pill			0.919		
Yes	212 (76.8)	213 (77.2)		0.98 (0.65–1.47)	0.920
No	64 (23.2)	63 (22.8)		1 (Reference)	
Family history of diabetes in first degree relatives			<0.001		
Yes	29 (10.5)	9 (3.3)		3.22 (1.53–6.81)	0.002
No	247 (89.5)	267 (96.7)		1 (Reference)	

*P-value was obtained from student t-test for continuous variables and chi-square test for categorical variables. **P-value was obtained from the multiple logistic regression model that simultaneously included biological factors.

### Best-fit model predicting the strongest association between all factors and GDM

In the final best-fit model (Table 4), the variables of education, occupation, household income, residency in Beijing, alcohol consumption before pregnancy, and alcohol consumption during pregnancy were changed from significant to non-significant, and were removed from the best-fit model. In contrast, the ORs for passive smoking at home from the husband (OR = 1.52, p = 0.027), passive smoking in the workplace (OR = 1.71, p = 0.01), and a family history of diabetes in first degree relatives (OR = 3.07, p = 0.004), were unchanged from estimates of factors associated with GDM observed in the original model (Tables 1–3). As a result of these further tests of significance (Table 4), these three variables remained in the best-fit model of the stronger factors associated with GDM.

**Table 4:** Associated factors identified in backward stepwise logistic regression (best-fit) model.

Variables	*Estimate	Standard error	Wald test	p-value	**OR (95%CI)
Passive smoking at home	0.42	0.19	4.87	0.027	1.52 (1.05–2.20)
Passive smoking in the workplace	0.53	0.21	6.62	0.010	1.71 (1.14–2.56)
Family history of diabetes in first degree relatives	1.12	0.39	8.41	0.004	3.07 (1.44–6.55)

Variables entered into the model: education, occupation, income, marital status, residency in Beijing, living condition, passive smoking at home, passive smoking in the workplace, alcohol consumption before pregnancy, alcohol consumption during pregnancy, physical activity, sleeping hours, number of pregnancies, family history of  diabetes in first degree relatives. *Values are the estimated non-standardized regression coefficients. **OR indicates likelihood of GDM. Significant level p ≤ 0.05.

### Correlation matrix of related independent variables

The information about the strength of correlations between related independent variables is shown in Table 5. Strong correlations between socioeconomic factors such as education and occupation (r = 0.39, p < 0.0001), education and household income (r = 0.37, p < 0.0001), occupation and household income (r = 0.31, p < 0.0001), were observed (Table 5). A strong correlation between alcohol consumption before pregnancy and alcohol consumption during pregnancy (r = 0.69, p < 0.0001) was found (Table 5). In addition, alcohol consumption before pregnancy and passive smoking at home (r = 0.17, p < 0.0001), alcohol consumption during pregnancy and passive smoking at home (r = 0.14, p < 0.001), were also significantly correlated as shown in Table 5.

**Table 5:** Correlation coefficients (r) from Pearson correlation analysis between socioeconomic, environmental and lifestyle factors, and biological factors.

	Education	Occupation	Income	Marriage	Residency	Living conditions	Passive smoke at home	Passive smoke at work	Drinking before pregnancy	Drinking during pregnancy	Physical activity	Sleep hours	Number of pregnancies	Family history diabetes
Education	1.00													
Occupation	**0**.**39** **(**<**0**.**0001)**	1.00												
Income	**0**.**37** **(**<**0**.**0001)**	**0**.**31** **(**<**0**.**0001)**	1.00											
Marriage	0.03 (0.466)	−0.04 (0.371)	0.02 (0.599)	1.00										
Residency	0.07 (0.105)	−0.01 (0.928)	0.09 (0.027)	−0.09 (0.041)	1.00									
Living conditions	**0**.**25** **(**<**0**.**0001)**	**−0**.**19** **(**<**0**.**0001)**	**−0**.**29** **(**<**0**.**0001)**	0.01 (0.739)	**−0**.**16** **(**<**0**.**001)**	1.00								
Passive smoke at home	−0.03 (0.468)	−0.02 (0.680)	−0.04 (0.397)	0.02 (0.576)	0.08 (0.066)	0.02 (0.692)	1.00							
Passive smoke at work	0.10 (0.021)	**0**.**12** **(0**.**007)**	**0**.**12** **(0**.**004)**	−0.04 (0.333)	0.02 (0.646)	**0**.**13** **(0**.**003)**	0.07 (0.106)	1.00						
Drinking before pregnancy	0.10 (0.018)	0.09 (0.044)	**0**.**16** **(**<**0**.**001)**	0.01 (0.764)	0.01 (0.884)	−0.09 (0.026)	**0**.**17** **(**<**0**.**0001)**	0.08 (0.061)	1.00					
Drinking during pregnancy	0.12 (0.004)	0.09 (0.033)	**0**.**14** **(0**.**001)**	−0.04 (0.396)	0.02 (0.570)	−0.10 (0.022)	**0**.**14** **(0**.**001)**	0.04 (0.307)	**0**.**69** **(**<**0**.**0001)**	1.00				
Physical activity	**0**.**14** **(0**.**001)**	0.08 (0.077)	0.03 (0.461)	−0.06 (0.170)	0.02 (0.665)	−0.09 (0.028)	0.02 (0.611)	0.001 (0.969)	0.05 (0.239)	0.06 (0.151)	1.00			
Sleep hours	0.04 (0.398)	0.099 (0.020)	−0.06 (0.195)	−0.07 (0.106)	**0**.**12** **(0**.**004)**	−0.10 (0.014)	−0.02 (0.721)	0.04 (0.349)	−0.001 (0.991)	−0.04 (0.396)	0.01 (0.899)	1.00		
Number of pregnancies	**−0**.**18** **(**<**0**.**0001)**	−0.11 (0.011)	−0.01 (0.707)	−0.01 (0.799)	**0**.**15** **(**<**0**.**001)**	0.02 (0.705)	0.08 (0.05)	0.04 (0.407)	0.04 (0.311)	0.01 (0.829)	−0.11 (0.010)	−0.02 (0.689)	1.00	
Family history diabetes	**0**.**15** **(**<**0**.**001)**	0.02 (0.631)	0.08 (0.060)	−0.01 (0.821)	0.11 (0.010)	−0.08 (0.058)	0.01 (0.847)	−0.001 (0.968)	0.03 (0.496)	0.03 (0.451)	−0.01 (0.808)	−0.01 (0.728)	−0.03 (0.421)	1.00

(figures in parentheses are p values, bolded figures when p < 0.01).

## Discussion

In this study, we examined the effects of socioeconomic, environmental and lifestyle factors associated with GDM in a population of pregnant Chinese women in Beijing. The results showed that compared to pregnant women without GDM, those developing GDM were more likely to be exposed to passive smoking both at home and in the workplace. Women with GDM were also more likely to have a family history of diabetes in first degree relatives.

In 2015 the Chinese Center for Disease Control and Prevention released the Chinese Adults Tobacco Survey Report^18^. This report showed that about 52.1% of Chinese men and 2.7% of Chinese women (age ≥15 years), corresponding to more than 316 million people, were current smokers. While the cigarette smoking rate of men is very high, this also puts women at a high risk for problems due to passive smoking. A previous study in Tianjin^19^ indicated that passive smoking is an independent risk factor associated with GDM. Our findings also indicate that pregnant women exposed to passive smoking both at home (1.5 times higher) and in the workplace (1.7 times higher) are more likely to be associated with the risk of GDM when comparing to healthy pregnant women.

China is the largest tobacco producer and consumer in the world^20,21^. Chinese national legislators have actively started the process of national bans on smoking in public and workplaces since 2014^22^. However, because of alleged significant interference from the tobacco industry, few effective legislative, executive, administrative or other measures designed to protect all people from exposure to tobacco smoke have been implemented at any governmental level^23,24^. The passive smoking problem in China is widespread and may impact health in this nation^25,26^.

Previous reports examined the relationship between passive smoking and lung cancer, coronary heart disease, respiratory diseases, and found serious side effects especially on infants and children^27^. Tobacco use is a leading risk factor for premature mortality and disability from non-communicable diseases in China^22^. According to the 2010 GATS survey^28^, women are primarily affected because the prevalence of passive smoking among non-smoking women is more than 63.9% in those exposed at home and 53.2% exposed in the workplace. Passive smoking is highly prevalent among women and has been a major concern in China^29^.

 Beijing Chaoyang district where our present survey was conducted is a famous business district where many white-collar workers live and work. This includes women who may be frequently exposed to passive smoking at their home and in the workplaces during their working hours and social activities. Because of the high proportion and rate of smoking among Chinese men, it may be difficult for women to avoid passive smoking because of the social environment around women^28^. To control cigarette smoking, it would be beneficial if the Chinese Government established strict state-level legislation on tobacco control, increased tobacco taxation, increased support for smoking cessation, legislated the prohibition of tobacco advertising, printed eye-catching warnings on cigarette packs, and implemented smoking bans in public areas^30^. It may also be important for the government to establish a new national tobacco control bureau that is independent of the tobacco industry^22^. The Chinese Government could also develop corresponding strategies to implement WHO FCTC’s recommendations^31^, so as to reduce the high burden of chronic non-communicable diseases.

Based on the findings of our study, we would also suggest that pregnant women who are exposed to passive smoking might be a special target group for prevention measures. These women may not know that passive smoking can seriously harm their health and that of their children in the same way as active smoking. From a public health perspective, our recommendations are for anti-smoking campaigns and tobacco control education to change the surrounding environment for these women who are at a high risk to develop GDM.

The percent of women consuming alcohol during pregnancy has been reported as a serious problem in other countries, such as 32.5% in Congo^32^, 4.4% in India^33^, 10% in USA and 17%-25% in Canada^34^. In our study, little evidence was observed for an association between alcohol consumption before/during pregnancy and the development of GDM in Chinese women. We did find significant results between alcohol consumption before/during pregnancy and GDM from the logistic regression analysis (Table 2). However, the significance was not strong enough to retain these two variables in the best-fit model (Table 4). As shown in Table 5, the reason may be due to the interactions between the related independent variables such as alcohol consumption before pregnancy and alcohol consumption during pregnancy (r = 0.69, p < 0.0001), alcohol consumption before pregnancy and passive smoking at home (r = 0.17, p < 0.0001), alcohol consumption during pregnancy and passive smoking at home (r = 0.14, p < 0.001). Correlations between these independent variables could influence the impact of alcohol consumption variables on GDM, and this removed these weaker variables from the final best fit model (Table 4). Further investigations are needed before definitive conclusions can be made about the possible effects of these alcohol consumption factors on GDM in Chinese women.

Socioeconomic status (SES) reflects the positions and monetary stability that individuals hold within the structure of the society. Research has shown that higher SES may be correlated with better health and longer life^35^. However, the relationship between socioeconomic factors and GDM were inconsistent from the previous studies. The studies in Italy^36^, the Netherlands^37^, and Australia^38^ suggested that lower SES was associated with GDM, while a study in India^39^ found higher SES was associated with GDM. In China, a study in Wuhan^40^ suggested higher educational lever was inversely associated with risk of GDM, while other studies in Beijing^17^ and Chongqing^41^ indicated no association between SES and GDM diagnosis. Consistent with these results in Beijing and Chongqing, our study also suggests no association between socioeconomic factors and GDM. The reason might be due to the correlations between socioeconomic variables such as education and occupation (r = 0.39, p < 0.0001), education and household income (r = 0.37, p < 0.0001), occupation and household income (r = 0.31, p < 0.0001), as shown in Table 5. These correlations could influence the variables that remain in the final best fit model (Table 4). Another reason might be the study sample selected from the two hospitals located in Beijing Chaoyang district which is a business and migrant focused area in Beijing. In 1958, China’s government established a “hukou” system to prevent rural to urban migration by requiring people to stay in the area where they were registered^42^. Under this system, rural citizens have no access to social welfare in the cities, even though they may live and work there^42^. Most white-collar workers who live in Beijing Chaoyang District are highly educated migrants from other Chinese provinces. Because housing and living standards in the Chaoyang District are extremely expensive and the urban life is very busy and stressful, most migrant women may live under this social and psychological stress, especially when they are pregnant. Although our study indicates that there is no association between socioeconomic factors and the development of GDM, the sample of this study could not represent the general population of pregnant women in Beijing. Because of this, the underlying causal mechanisms for our data may warrant further investigations before conclusions can be made.

Consistent with previous investigations^15,17^, our present data confirm the findings that pregnant Chinese women with a family history of diabetes in first degree relatives were at higher risk (3 times) of developing GDM than pregnant women without this history. We also found that this association is not influenced by other confounding factors. Screening and early identification of these possible risk factors in pregnant patients would be helpful and cost-effective in planning maternal health services and providing high quality prenatal care to women who may develop GDM.

Certain limitations should be considered in interpreting the results of our current study. First, the sample we have drawn was from Chaoyang district, the business center in urban Beijing. Most of the women living in this area are from migrant families with higher socioeconomic background, so this study sample could not represent the general population of pregnant women in Beijing. Second, case-control studies offer only hints as to the causative factors that may lead to the development of GDM, and the use of terms such as “prediction” and “risk” do not imply causal or temporal relationships. Third, retrospective self-reporting of prenatal complications or lifestyle factors may be susceptible to recall bias, and variables such as tobacco and alcohol exposure may also be susceptible to recall bias. Future studies may be proposed to address these limitations.

In summary, findings from this case-control study suggest that significant factors such as passive smoking at home and in the workplace, and having a family history of diabetes mellitus are associated with development of GDM in Chinese women in Beijing. Development of diabetes in pregnancy may be prevented through public health intervention and lifestyle modification, but the adoption of a healthy lifestyle requires individual behaviour changes, and most importantly, may involve changes in the social environment. Public health strategies that focus on passive smoking risks and developing areas with 100% smoke free environments, could help to protect pregnant women at home and in the workplace. These proposals would be important and positive steps to approach solutions to this environmental tobacco smoke problem.

## Methods

### Study population

**Figure 1:**
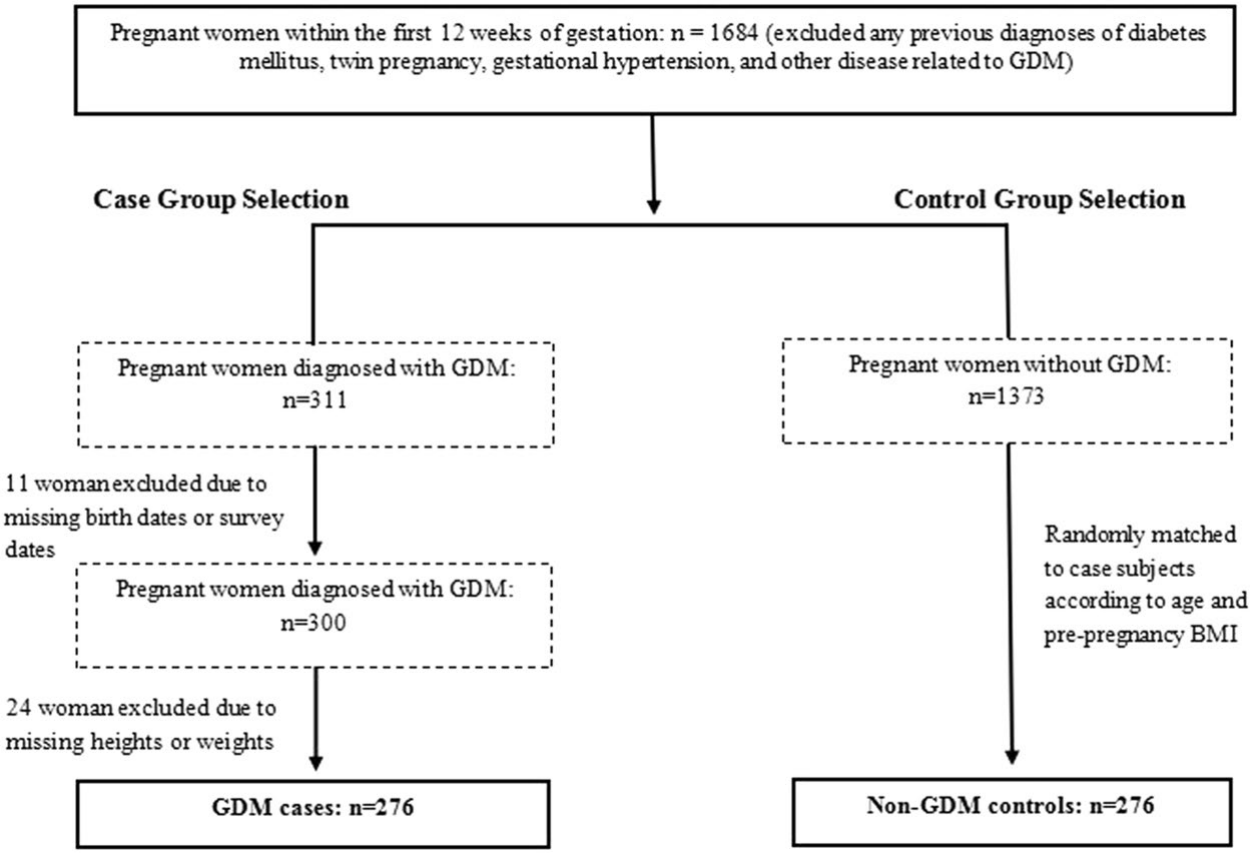
Flow chart illustrating the recruitment of GDM cases and controls.

This study was conducted from January 2012 to June 2014, and designed as a one-on-one matched pair case-control study. A total of 1684 pregnant women were recruited from the Beijing Chaoyang District Hospital of Maternal and Child Health and Beijing Chuiyangliu Hospital in Beijing, during their initial prenatal visit within the first 12 weeks of gestation (range from 7 to 12 week). The exclusion criteria for recruiting included pregnant women with previous diagnoses of diabetes mellitus, twin pregnancy, gestational hypertension, or other diseases that are already known as risk factors for GDM (Figure 1). In this population of 1684 pregnant women, 311 women were clinically diagnosed with GDM (prevalence 18.5%), and 11 of these were excluded from the study because of missing birth dates or survey dates. As a result, 300 pregnant women with GDM qualified for the group and pair matching analyses. According to the statistical literature^43^, this matching refers to selection of control subjects and GDM case subjects based on specific criteria of similarity: age and pre-pregnancy BMI. In this study, we used this pair matching method to determine the comparable groups: 276 pregnant women with GDM were then identified as the case group, and 276 pregnant women without GDM were randomly matched for each comparison (Figure 1).

Data were collected by utilizing carefully designed questionnaires that were completed by obstetricians and participants during the initial prenatal visit. Ethics approval for this study was obtained from the Ethics Committee of Beijing Chaoyang District Hospital of Maternal and Child Health and Beijing Chuiyangliu Hospital. All procedures and methods were performed by the authors in accordance with the approved guidelines. All participants provided written informed consent for the study, and blood samples and medical records were then collected.

### Diagnostic criteria of GDM

A 1-hour glucose challenge test was used to screen pregnant women for GDM between 24 and 28 weeks of gestation at Beijing Chaoyang District Hospital of Maternal and Child Health and Beijing Chuiyangliu Hospital. Non-fasting venous blood was taken 60 minutes after the ingestion of 200 ml of 25% glucose solution to measure plasma glucose (PG). Women with PG ≥7.8 mmol/L were referred for this standard 75-gram 2-hour oral glucose tolerance test^15^: After overnight fasting of at least 8 hours, the women ingested 300 ml of 25% glucose solution in the morning, and venous blood was drawn at fasting, 1 hour and 2 hours after the glucose load. All blood samples were tested using an automatic analyzer (Toshiba TBA-120FR, Japan). The International Association of Diabetes Study Group criteria were used for diagnosis of GDM as defined by one of the following: fasting plasma glucose ≥5.1 mmol/L and/or 1-hour PG ≥10.0 mmol/L and/or 2-hour PG ≥8.5 mmol/L^44^.

### Matched factors

To address important confounding factors such as advanced pregnancy ages and pre-pregnancy BMI being associated with an increased prevalence of GDM^15,17,45–47^, we designed pair-matches on age and pre-pregnancy BMI for the control vs. GDM subjects in this study. Body height and weight were measured with a beam balance scale (RGZ-120, Jiangsu Suhong Medical Instruments Co., China). Body weight at the initial prenatal visit was treated as pre-pregnancy weight due to only small weight gains during the first 12 gestational weeks^48,49^. BMI was calculated as weight in kilogram divided by the square of body height in meter.

### Socioeconomic factors

Socioeconomic status (SES) was assessed for education, occupation, and monthly household income. Three levels were assigned for education: low (≤9 years compulsory education), middle (9–12 years high school), high (>12 years college and above)^40^. Occupation was defined as four categories: housewife, manual labor, office worker, or other type of job^50^. Monthly total household income was divided into four groups: <3000 Chinese Yuan, 3000–5999 Chinese Yuan, 6000–8999 Chinese Yuan, or >9000 Chinese Yuan (1.00 US Dollar ≈ 6.00 Chinese Yuan in 2014). In addition to these three well-known SES indicators, we measured the following factors which might also reflect sociodemographic structures and social functions affecting the women’s health. These factors included belonging to either the Chinese Han or minority ethnic group, marital status including married or unmarried (single/divorced/widowed), length of residence in Beijing (>5 years or ≤5 years), and living conditions (home owner, renter, living with parents, other).

### Environmental and lifestyle factors

Environmental tobacco smoke (ETS) was defined as a non-smoker being exposed to another person’s tobacco smoke for at least 15 min daily for more than one day per week^20,21^. Passive smoking at home from the husband, passive smoking from another family member, and passive smoking in the workplace were included as ETS variables^51,52^. Habitual smoking before pregnancy or during pregnancy was defined as continuously smoking one or more cigarettes per day for at least 6 months before pregnancy, or smoking one or more cigarettes per day during pregnancy^53^. Alcohol consumption habits were assessed using two questions: (1) “Have you consumed any kind of alcohol in the past 6 months before pregnancy?”; (2) “Have you consumed any kind of alcohol during your pregnancy^32,33^?” To report physical activity, the participants were asked the following two questions: (1) “During the last 30 days, on how many days were you physically active at least 60 minutes per day in a week?” The answers were divided into the following: physical activity <3 days in 1 week, or physical activity ≥3 days in 1 week. (2) “During the last 30 days, have you participated at least 2 times a week in any kind of sports such as swim/dance/playing ball?” The participants were also asked about how many hours they spent viewing TV and sleeping daily. We also included asking about the daily intake of fruits and sugar-sweetened soft drinks, which may be also associated with GDM^13,54^.

### Biological factors as the covariates

The participants completed the questionnaires including pregnancy-related information, such as menstrual cycle, number of previous pregnancies, number of live births, number of miscarriages/abortions, and if they had been taking any oral contraceptive pills. The participants were also asked if they had a family history of diabetes mellitus in their first-degree relatives.

### Statistical analysis

All analyses were performed using the SAS 9.4 statistical software package (SAS Institute Inc., Cary, NC, USA). In the first step, descriptive statistics were used to profile socioeconomic, environmental and lifestyle factors, and biological factors of all study subjects. GDM cases and controls were compared using student t-test for continuous variables and chi-square test for categorical variables^55^. To assess the association between independent variables and the dependent variable of GDM, multiple logistic regression analysis was performed with all independent variables simultaneously included in the same model^56^.

The second step involved using the conditional logistic regression analysis with backward stepwise procedures. This was performed based on the maximum partial likelihood estimates to construct the final best-fit model^57^, and to identify the predictors of risk for GDM among all independent variables. This model retests those variables at p less than 0.2 to determine which variables have the strongest significance. The backward stepwise method^58^ starts with all variables in the model, then removes the variable with the least statistically significant until all remaining variables have a significant p-value. We estimated odds ratios (ORs) and 95% confidence intervals (CIs) for differing levels of exposure. All statistical tests were considered to be significant at an alpha level of 0.05 on a two-tailed test.

In the last step of analyses, the correlations between related independent variables such as socioeconomic factors, environmental/lifestyle factors, and biological factors were examined using the Pearson correlation test.

### Data availability

The data analyzed during the current study are not publicly available because they include personal identifiers and medical information that cannot be released, but are available from the corresponding author on reasonable request.
